# Tebuconazole Induces ER-Stress-Mediated Cell Death in Bovine Mammary Epithelial Cell Lines

**DOI:** 10.3390/toxics11040397

**Published:** 2023-04-21

**Authors:** Won-Young Lee, Ran Lee, Hyun-Jung Park

**Affiliations:** 1 Department of Livestock, Korea National University of Agriculure and Fisheries, Jeonju-si 54874, Republic of Korea; 2 Department of Animal Biotechnology, College of Life Science, Sangji University, Wonju-si 26339, Republic of Korea

**Keywords:** tebuconazole, fungicide, milk production, bovine, mammary glands, endoplasmic reticulum stress

## Abstract

Tebuconazole (TEB) is a triazole fungicide used to increase crop production by controlling fungi, insects, and weeds. Despite their extensive use, people are concerned about the health risks associated with pesticides and fungicides. Numerous studies have defined the cellular toxicity of triazole groups in pesticides, but the mechanisms of TEB toxicity in bovine mammary gland epithelial cells (MAC-T cells) have not yet been studied. Damage to the mammary glands of dairy cows directly affects milk production. This study investigated the toxicological effects of TEB on MAC-T cells. We found that TEB decreases both cell viability and proliferation and activates apoptotic cell death via the upregulation of pro-apoptotic proteins, such as cleaved caspases 3 and 8 and BAX. TEB also induced endoplasmic reticulum (ER) stress via the upregulation of Bip/GRP78; PDI; ATF4; CHOP; and ERO1-Lα. We found that TEB induced mitochondria-mediated apoptotic MAC-T cell death by activating ER stress. This cell damage eventually led to a dramatic reduction in the expression levels of the milk-protein-synthesis-related genes LGB; LALA; CSN1S1; CSN1S2; and CSNK in MAC-T cells. Our data suggest that the exposure of dairy cows to TEB may negatively affect milk production by damaging the mammary glands.

## 1. Introduction

Several pesticides are active ingredients belonging to the triazole group and control pests such as insects, fungi, and weeds [1]. The triazole group of pesticides has been widely used in agriculture since the 1970s. In particular, the triazole group exhibits strong antifungal activities [2]. Despite its usefulness, triazoles can be toxic to non-target organisms. For example, cyproconazole (50–250 µM) is associated with increased risks during zebrafish embryo development, and exposure to propiconazole in a *Xenopus Tropicalis* model results in increased toxicity to the hepatic and reproductive systems during puberty [3,4]. Tebuconazole (TEB) is an important fungicide that belongs to the triazole family and effectively protects plants and crops against fungal diseases in the agricultural sector [5]. TEB acts by interfering with the biosynthesis of ergosterol, which is a major product of sterol biosynthesis in fungi, and leads to the inhibition of cell growth or cell death in fungi [6]. The use of TEB in agriculture has continued to increase since 1992, and its use has been extended to various ingredients such as fruits, soybeans, rice, vegetables, grapes, corn, and hay. The production of crops and animal-derived foods has become highly dependent on the use of pesticides. Due to its prevalent use, pesticide presence and accumulation has been reported in several food and products [7,8]. Previously, Zuburod et al. reported that TEB exists in stream and surface water and its concentration in surface water has reached 175–200 μg/L [9].

Several studies have reported toxic effects of TEB in rodents. The results show that TEB induces developmental disturbances, reproductive dysfunction, immunological abnormalities, hepatotoxicity, and nephrotoxicity [10,11]. In rodents, TEB induces cardiac toxicity via ROS-dependent apoptotic cell death by inducing DNA damage and mitochondrial apoptotic pathways [12,13]. TEB damages the structure and function of the placenta and induces hypertrophy of the placenta in female rats. In utero exposure to TEB causes proliferation of fetal Leydig cells and increases testosterone production during puberty by directly inhibiting CYP19A1 activity in the testes [14,15].

Chemicals such as pesticides in animal feeds may be transferred to the organs or tissues of livestock during feeding. Therefore, TEB can accumulate in living animals, and it may be related to reduced livestock productivity. The bovine mammary gland consists of fibroblasts, adipocytes, and epithelial and myoepithelial cells. Milk is produced in the udder by mammary epithelial cells (MECs) in the mammary glands. The number of MECs in mammary glands and their secretory activity are critical factors for milk production. Damage to MECs, including apoptosis, affects milk synthesis and production [16]. Residues of TEB in feed may be harmful to livestock, and pesticides are widely present in the feed of dairy cows [17]. Several studies have reported the effects of pesticides on the bovine. Among pesticides, diflubenzuron inhibits the proliferation of bovine MECs via reactive oxygen stress (ROS)-mediated mitochondrial dysfunction [18]. The herbicide pendimethalin also inhibits cell proliferation via the induction of endoplasmic reticulum (ER) stress and disrupts mitochondrial membrane potential in bovine MECs [19]. In addition, another herbicide, aclonifen, induces MEC death by disrupting calcium homeostasis and ROS production [20].

Despite growing concerns about the toxicity of TEB impacting human and animal health, the effect of TEB on cattle remains unclear. In this study, we evaluated whether TEB is toxic to bovine mammary epithelial cells and conducted research on the mechanism of toxicity.

## 2. Materials and Methods

### 2.1. Cell Culture and Treatment

Immortalized bovine mammary alveolar cells (MAC-T) were cultured in Dulbecco’s Modified Eagle’s medium with 10% fetal bovine serum and 1% penicillin–streptomycin, in a humidified atmosphere of 5% CO_2_ at 37 °C.

Tebuconazole, TEB (Sigma-Aldrich, St. Louis, MO, USA), was dissolved in dimethyl sulfoxide (DMSO) to prepare a 1 M stock solution, which was diluted to the desired concentration using cell culture medium prior to cell culture.

### 2.2. Cytotoxicity Tests

First, the cytotoxicity induced by treatment with the test substances was evaluated by performing a 3-(4,5-dimethylthiazole-2-yl)-2,5-diphenyl tetrazolium bromide (MTT) assay using the EZ-Cytox Viability Assay Kit (Daeil Lab Services Co., Seoul, Republic of Korea, #EZ1000) according to the manufacturer’s instructions. The TEB half-maximal inhibitory concentration (IC_50_) in MAC-T cells was calculated (LC_50_ = 230 µM). Cells were seeded in 96-well plates at a density of 2 × 10^3^ cells/well in culture medium and cultured for 12 h. Next, 0–300 µM TEB was added in cultured medium and incubated for additional 24 h. Consequently, reagent was added from assay kit, and wells were incubated for 1h in the cell culture incubator. Absorbance was measured at 490 nm using an epoch spectrophotometer (Bio Tek, Winooski, VT, USA). MAC-T cell images were obtained using a microscope (Nikon E−800; Nikon, Tokyo, Japan) after 24 h with 0–200 µM TEB treatment.

### 2.3. Flow Cytometry

Annexin V-FITC staining was performed using a cell death apoptosis kit (Thermo Fisher Scientific, Inc., Waltham, MA, USA) according to the manufacturer’s instructions as previously described [21]. Briefly, cells were seeded in 6-well plates and treated with TEB for 24 h. Cells were harvested and washed with PBS for staining with Annexin V-FIC and PI in the dark. Annexin V-positive cells were detected by flow cytometry (CytoFLEX; Beckman Coulter, Inc., Miami, FL, USA). Additionally, JC-1 staining (Biotium Inc, Fremont, CA, USA) was carried out to detect mitochondrial membrane potential (*∆Ψm*) of MAC-T cells. The cells were harvested after culturing for 24 h with TEB and washed with ice-cold PBS. Then, cells were resuspended in 500 μL of 1X jC-1 reagent working solution and incubated at 37 °C for 15 min, before staining according to the manufacturer’s instructions. The stained cells were then washed with PBS and analyzed by flow cytometry (CytoFLEX, Beckman Coulter, Inc., Miami, FL, USA), and cell images were collected using a microscope (Nikon E−800; Nikon, Tokyo, Japan).

### 2.4. Immunostaining

MAC-T cells were seeded on 18 mm glass coverslips (BD Biosciences, Franklin Lakes, NJ, USA) and treated with TEB concentrations of 0–200 µM for 24 h. After fixation with 4% paraformaldehyde at 18 °C for 15 min, the cells were permeabilized in 0.1% Triton X-100 in PBS solution (0.1% Triton X-100 in PBS) for 10 min at 18 °C. Then, samples were incubated with the primary antibody Ki-67 at 4 °C overnight followed by incubation with FITC-conjugated (anti-rabbit/anti-mouse) secondary antibody at room temperature for 1 h. The proliferation index was defined as the ratio of (the number of Ki-67-postive cells)/(the total number of cells) in each field.

### 2.5. RNA Isolation and Quantitative PCR

Total RNA was extracted using an RNeasy Mini Kit (Qiagen, Hilden, Germany) with on-column DNase treatment (Qiagen). Complementary DNA (cDNA) was synthesized by reverse transcription using a RevertAid Fist Strand cDNA synthesis kit (Thermo Scientific, Rockford, IL, USA). Quantitative real-time PCR (qPCR) was performed on a QuatStudio 1 instrument (Applied Biosystems, Foster City, CA, USA) using SYBR mixture (Thermo Fisher Scientific, Waltham, MA, USA). The reaction was carried out at 94 °C for 1 min, followed by 40 cycles at 94 °C for 10 s, 57 °C for 10 s, and 72 °C for 20 s, and quantification was performed by normalization to the endogenous GAPDH levels. Relative quantification (RQ) of the fold-change between the treatment and reference controls was determined as previously described (33396729). Table 1 presents the primers used in this experiment and those designed using Primer3 (http://frodo.wi.mit.edu accessed on 1 January 2021).

### 2.6. Western Blotting

MAC-T cells were harvested after culture with TEB treatment and lysed using RIPA lysis buffer (Thermo Scientific™, Rockford, IL, USA) containing a protease inhibitor mixture (Roche, Rotkreuz, Switzerland). The total protein content was quantified using a BCA assay kit (Thermo Fisher Scientific, Waltham, MA, USA). First, 30 micrograms of total protein was loaded onto 4–16% gradient SDS-PAGE gels (Bio-Rad, Hercules, CA, USA) and transferred to PVDF membranes using a transfer blotting system (Bio-Rad, Hercules, USA). Then, membranes were incubated for 16 h with primary antibodies in blocking solution (TBS with 0.1% tween-20 (TBST) + 1% bovine serum albumin) at 4 °C. The membranes were incubated with a horseradish peroxidase-conjugated secondary antibody (anti-mouse/rabbit antibody) for 1 h after washing with TBS. Western blots were visualized using Pierce ECL solution (Thermo Fisher Scientific, Rockford, IL, USA) and iBright™ Imaging Systems (Thermo Fisher Scientific, Inc., Waltham, MA, USA). *β*-actin was used as the normalization control, and the antibodies used for immunoblotting are listed in Table 2. Antibodies were purchased from Santa Cruz (Dallas, Texas, USA) and Cell signaling (Danvers, MA, USA).

### 2.7. Statistical Analysis

Data were expressed as mean ± standard error (SEM) of at least three independent experiments conducted in triplicate. Mean differences were evaluated using one-way analysis of variance (ANOVA), followed by Tukey’s post hoc test. All statistical analyses were conducted using the SPSS statistical package, version 15.0, for Windows (IBM Corp, Somers, NY, USA). Comparisons were considered statistically significant at * p < 0.05 and ** p < 0.01.

## 3. Results

### 3.1. TEB Induced MAC-T Cell Apoptosis and Toxicity

**Figure 1:**
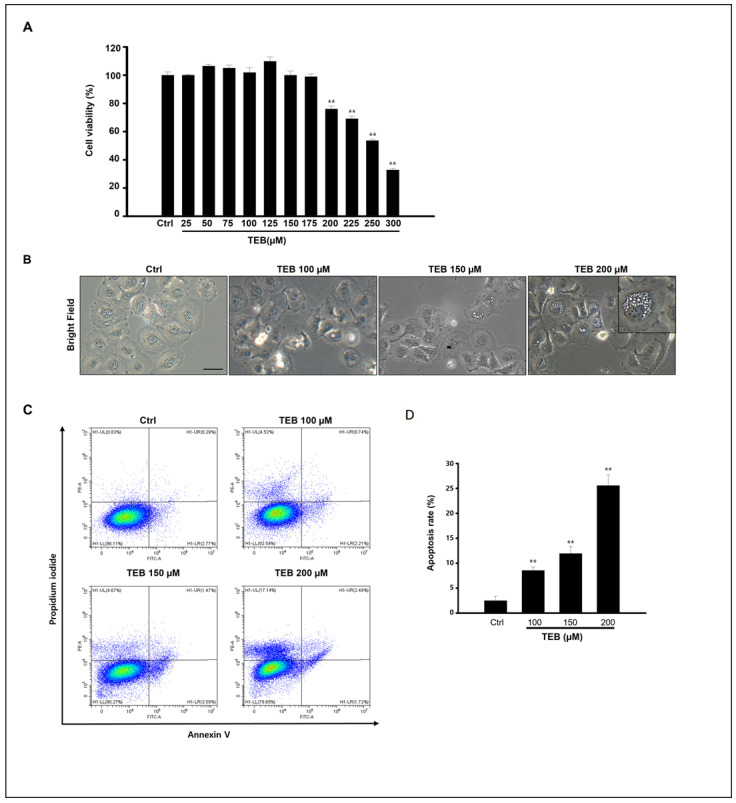
Effects of TEB on viability, proliferation, and apoptosis of MAC-T cells. (**A**) MAC-T cell viability assessed by MTT assay. DMSO, cells treated with TEB (0–300 μM). (**B**) Morphological images of the cells observed under a microscope after 24 h treatment. Cells were treated with 0–200 μM of TEB in culture. Scaler bar = 20 μM. (**C**) The apoptotic cell death of MAC-T cells by TEB exposure was analyzed by flow cytometry at different concentrations (0–200 μM). Apoptotic cell death was determined by Annexin V-FITC/PI staining. (**D**) The graph shows the apoptosis rate, all data are presented as the mean ± SD of three independent experiments, and significance levels between control and treated are shown as asterisks (n = 4, ** *p* < 0.001).

Cytotoxicity of TEB to MAC-T cells was investigated by measuring cell viability using an MTT assay. Cells were exposed to various concentrations (0–300 μM) of TEB for 24 h (Figure 1A). The percentage of cell viability decreased over the concentration of 200 μM in a dose-dependent manner (170–300 μM, IC_50_ value is 230 µM). The concentrations of TEB were selected from these data (0–200 μM). In Figure 1B, the morphological changes in MAC-T cells were distinctly observed in 150–200 μM of TEB-exposed MAC-T cells. TEB treatment induced vacuole formation adjacent to the nucleus in the cytoplasm of MAC-T cells. These vacuoles were first observed when MAC-T cells were treated with 150 μM of TEB for 24 h (Figure 1B). Next, to clarify the effect of TEB on cell death, Annexin V fluorescein isothiocyanate (FITC) and propidium iodide (PI) labeling was performed (Figure 1C). Early apoptotic cells were visualized using an Annexin V-FITC+/PI- staining pattern, whereas late apoptotic cells exhibited an Annexin V-FITC+/PI+ staining pattern. Our results show that approximately 20–25% of the cells were observed to be apoptotic after treatment with 200 μM TEB (Figure 1E). These results suggest that TEB induces apoptotic cell death in MAC-T cell cultures.

### 3.2. Anti-Proliferation Effect of TEB on MAC-T Cell Culture

**Figure 2:**
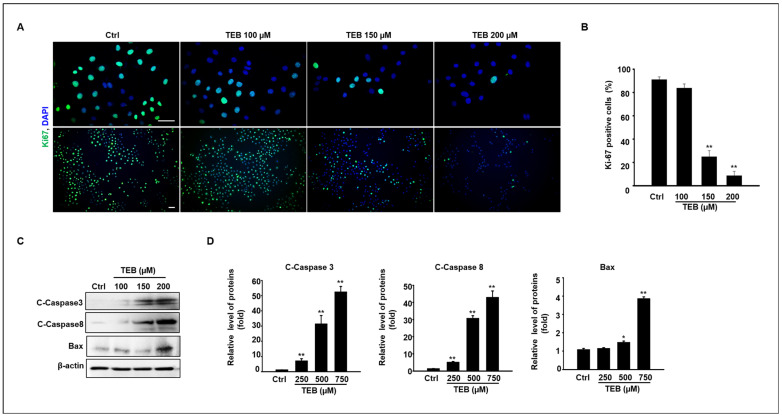
The effects of TEB on MAC-T cell proliferation and pro-apoptotic protein expression. (**A**) Immunofluorescence analysis of Ki-67 in MAC-T cells after culture with 0–200 μm TEB. Scale bar = 50 μm. (**B**) Quantification of Ki-67-positive cells (Green) with respect to total cells (Blue) (%). Data are presented as mean ± SD (n = 4, ** *p* < 0.001). (**C**) MAC-T cells were treated with the 0–200 μM TEB for 24 h, and then total protein was prepared and analyzed by immunoblotting. The protein expression levels of cleaved caspases 3 and 8, BAX, and β-actin in each experimental group. (**D**) The graph shows the densitometry analysis of protein bands normalized to β-actin. Data are presented as the mean ± SD (n = 4, * *p* < 0.01, ** *p* < 0.001).

To clarify the effect of TEB on cell proliferation, immunostaining was performed using a Ki-67 antibody (Figure 2A). Ki-67-positive cells (FITC-positive) decreased in TEB-exposed MAC-T cells in a dose-dependent manner. Figure 2B shows the percentage of Ki-67 out of total cells (%) in the active phase of the cell cycle and the significant decrease in Ki-67-positive cells when cells were cultured with 150–200 μM TEB (Figure 2B). Based on the results of cell viability and anti-proliferation assays, key proteins involved in the pro-apoptotic pathway, such as cleaved caspases 3 and 8 and BAX protein, were normalized using β-actin (Figure 2C). The results show that the expression levels of pro-apoptotic proteins increased in MAC-T cells after treatment with 150–200 μM TEB (Figure 2D).

### 3.3. TEB Induces Mitochondrial Dysfunction in MAC-T Cells

**Figure 3:**
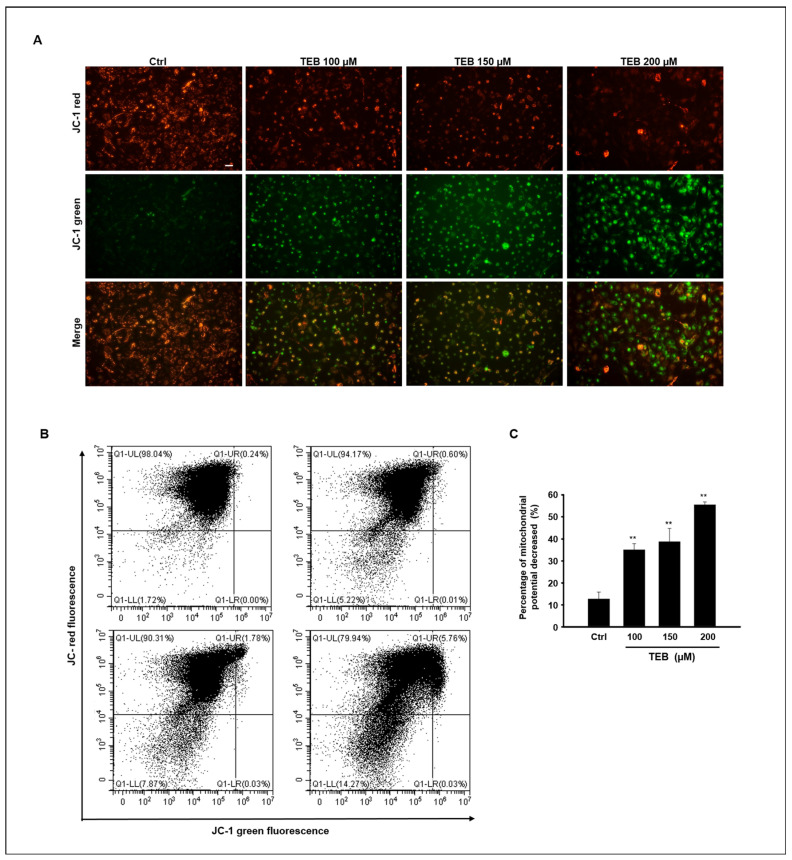
TEB induced loss of mitochondrial membrane potential in MAC-T cells. (**A**) Mitochondrial membrane potential in TEB-treated MAC-T cells was assessed using JC-1 staining. Red color indicates the accumulation of JC-1 aggregates, which indicates a normal mitochondrial membrane. Green color indicates JC-1 monomer with membrane depolarization. (**B**) Flow cytometry plots for MAC-T cells stained with JC-1 with TEB treatment (0–200 μM). (**C**) Relative ratios of green fluorescence (JC-1 monomer) represented as mitochondrial membrane potential (*∆Ψm*). Values represent the mean ± SD of three independent experiments (n = 3, ** *p* < 0.001 compared to the controls).

To detect potential mitochondrial dysfunction, MAC-T cells were treated with TEB. JC-1 staining was carried out to measure mitochondrial membrane potential (*∆Ψm*) in MAC-T cells (Figure 3A). JC-1 dyes form aggregates at high internal mitochondrial concentrations and emit red fluorescence, whereas at low membrane potentials, they are present as monomers and emit green fluorescence. The image shows that red fluorescence intensity decreased and green fluorescence intensity increased in the cells with increasing TEB concentration. These results suggest that TEB reduces mitochondrial membrane potential in MAC-T cells and is implicated in mitochondrial dysfunction (Figure 3B). The ratio of red to green fluorescence was standardized relative to that of the control. The relative *∆Ψm* ratio was reduced in a dose-dependent manner by TEB at concentrations of 100–200 μM (Figure 3C).

### 3.4. TEB Induced Apoptosis via ER Stress in MAC-T Cells

**Figure 4:**
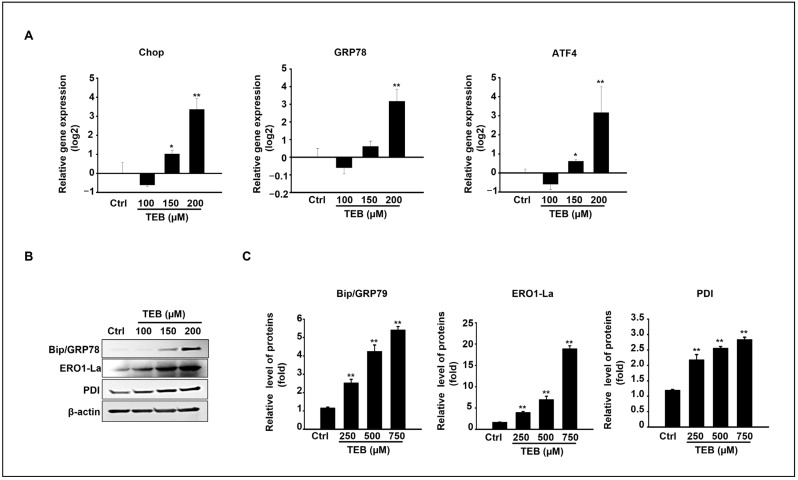
The expression of ER-stress-related genes and proteins in TEB-treated MAC-T cells. (**A**) mRNA expression levels of CHOP, Bip/Grp78, and ATF4 in MAC-T cells by qPCR after 24 h of TEB treatment. (**B**) Immunoblots for detecting Bip/Grp78, ERO1-Lα, PDI, and β-actin in response to the exposure to different concentrations of TEB. (**C**) The graphs present the results of densitometric analysis. Each protein band was normalized using β-actin. Data represent the mean ± SD of three independent experiments (n = 3, * *p* < 0.05, and ** *p* < 0.001 compared to the controls).

Based on our results, TEB triggers apoptosis and mitochondrial dysfunction in MAC-T cells. Additionally, the molecular mechanism was investigated based on ER-stress-related pathways in MAC-T cells cultured with TEB. The gene and protein expression levels of ER-stress-signaling molecules were evaluated in cells exposed to 0–200 μM TEB. Transcriptional levels of CHOP, GRP78, and ATF4 were dramatically increased at the highest concentration of TEB (200 μM) (Figure 4A). Consistent with the gene expression results, TEB treatment upregulated ER-stress-related protein levels (Figure 4B). Key ER-stress-related protein levels such as BiP/GRP78, PDI, and ERO1-Lα were statistically elevated in 150–200 μM TEB-treated samples compared to those of the control in a dose-dependent manner (Figure 4B,C).

### 3.5. TEB Regulated the Expression of Milk-Protein-Synthesis-Related Genes and Inflammatory Genes in MAC-T Cells

**Figure 5:**
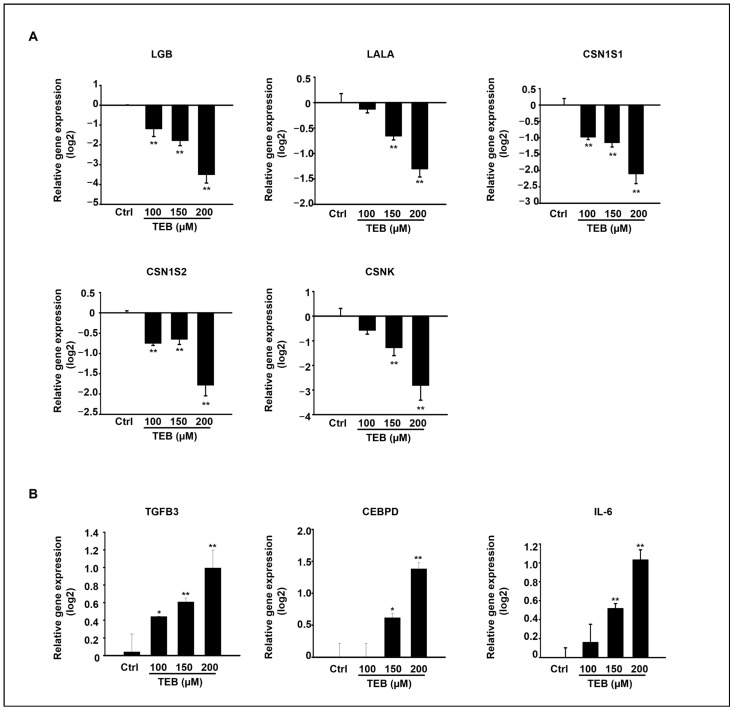
The expression of milk-protein-synthesis-related genes in TEB-treated MAC-T cells. (**A**) mRNA expression levels of LGB, LALA, CSN1S1, CSN1S2, and CSNK, and (**B**) the gene expression levels of inflammatory genes TGFB3, CEBPD, and IL-6 in TEB-exposed MAC-T cells by qPCR. The graph represents the mean ± SD of three independent experiments (n = 4 * *p* < 0.05, and ** *p* < 0.001 compared to the controls).

A previous study reported that both αs1-casein (CSN1S1) and β-casein (CSN1S2) genes were expressed in MAC-T cells [22]. The effect of ER-stress-induced apoptosis on the expression of milk-protein-synthesis-related genes was evaluated in MAC-T cells. The expression levels of milk-protein-synthesis-related genes, such as LGB, LALA, CSN1S1, CSN1S2, and CSNK, were significantly decreased in TEB-exposed MAC-T cells 24 h after treatment in a dose-dependent manner (Figure 5A). Additionally, the expression of inflammation-related genes TGFB3, CEBPD, and IL-6 dose-dependently increased in MAC-T cells after TEB treatment (Figure 5B).

## 4. Discussion

Fungicides and pesticides play critical roles in improving crop yields and reducing animal and human diseases worldwide. Despite these advantages, concerns about health risks such as food contamination and various environmental issues are gradually increasing [23,24]. TEB belongs to the triazole group, which is a class of fungicides associated with increased toxicity to various organs in the body, such as the liver [25], nervous system, colon [26], reproductive system [27], and heart [28].

In the present study, we evaluated the toxic effects of TEB on bovine mammary gland epithelia. The results show that TEB negatively affected MAC-T cell survival and proliferation and upregulated the expression of pro-apoptotic proteins, such as cleaved caspases 3 and 8 and Bax. Apoptosis is a process of programmed cell death and is a type of self-destruction mechanism involving a variety of biological events. Regulation of apoptotic cell death contributes to various diseases, cell growth, and tumor regression [29]. Typically, there are two apoptotic pathways, intrinsic and extrinsic. The intrinsic pathway is mitochondria-dependent and involves pro- and anti-apoptotic Bcl-2 family members; the extrinsic pathway consists of a cell surface TNF-related family of receptors, which is death-receptor-dependent [30].

Mitochondria, death receptors, and ER are the three major elements of the apoptotic pathway [31]. ER stress can activate either the intrinsic or the extrinsic pathway of apoptosis [32]. Mitochondria act as the center of the apoptotic pathway by providing many factors that can induce caspase activation and chromosome fragmentation [33]. In our study, TEB treatment induced caspase activation and mitochondrial dysfunction in MAC-T cell cultures, indicating that intrinsic pathways are involved in the induction of apoptosis. Several studies have demonstrated that cell death is induced via various pathways following TEB exposure. In some studies, TEB induced ROS-mediated cell death via mitochondrial dysfunction in human colon carcinoma, cardiac, and hepatic cells [13,34,35]. In other studies, TEB induced apoptosis via ROS in the kidney and liver [36,37], similar to previous studies. Among these previous studies, Othmène demonstrated not only ROS-mediated cell death but also ER-stress-dependent apoptotic cell death in HCT 116 cell culture after TEB treatment [26]. Interestingly, our results show that ER-stress-related factors such as GRP79, ATF4, CHOP, PDI, and ERO-La were dramatically increased in TEB-treated MAC-T cells, similar to the results of a previous study [26].

In the early stage of the ER stress response, ATF activates the pro-survival pathway [38], and ATF4 initiates the expression of the CHOP protein, which plays an important role in ER-stress-induced apoptosis [39]. ER-stress-induced apoptosis occurs via two key pathways: the C/EBP homologous protein (CHOP)/GADD153 pathway [40] and the IRE1/ASK1/JNK pathway [41]. Our study indicates that TEB may induce ER stress through the C/EBP homologous protein (CHOP)/GADD153 pathway, leading to apoptosis. In an in vitro MAC-T cell culture, Fu reported that zearalenone (ZEA), a common mycotoxin, reduced cell viability and increased intracellular ROS concentration, mitochondrial dysfunction, ER stress, and expression levels of apoptosis-related genes in a dose-dependent manner [42]. Although ZEA is not a fungicide, its toxic mechanism of apoptosis induction is similar to that mentioned above.

Bovine MECs produce and secrete dairy milk containing casein, milk fat, and various soluble components. Casein proteins are synthesized in MECs and released by exocytosis. Mammary gland epithelial secretory cells (MESCs) can regulate the secretory pathway of casein [43]. Interestingly, casein genes are expressed in MAC-T cells [22,44]. Based on these results, changes in milk-synthesis-related gene expression in MAC-T cells after TEB treatment were investigated. The mRNA levels of the major milk protein synthesis genes CSN1S1, CSN1S2, CSNK, LALA, and LGB were distinctly reduced in MAC-T cells by TEB. These results are likely due to MAC-T cell death and damage caused by ER stress induced by TEB exposure.

The amount of milk production and quality of milk produced are the most important indicators of household income in dairy farms. Healthy MECs are essential for optimal milk production. Nevertheless, excessive use of fungicides damages the MECs of crop-fed dairy cows, and the damaged MECs cannot produce optimal milk.

Only one study has reported accumulation of TEB in the body. In humans, Orelemans investigated the accumulation of TEB in urine samples. In total, 6 volunteers received a single oral dose of TEB (1.5 mg) for 1 h. Urine samples were collected 48 h after administration. The mean recovery of TEB metabolite hydroxyl-tebuconazole (TEB-OH) in urine during 48 h was 38% ± 16% [45]. Although there have been no previous studies on the accumulated TEB concentration in the bodies of dairy cows, the main staple diet of dairy cows is crops. Therefore, they were likely exposed to and accumulated high levels of pesticides and fungicides.

Additionally, our results show that TGFB3, CEBPD, and IL-6 were highly expressed in TEB-exposed MAC-T cells compared to the controls. This result also suggests a possible link between ER stress and gene expression in bovine mammary gland epithelia induced by TEB, although only mRNA levels were investigated in these studies. In mice astrocytes, Sanchez et al. noted that ER-stress-induced production of IL-6, which is an immune response cytokine, requires the PERK and JAK1 pathways but not IRE1 or nuclear factor-κB (NF-κB) signaling [46]. TGFB3 is also an immune response gene that crosstalks with IL-6. For example, IL6 induced TGFB3 production in cardiac fibroblasts and enhanced TGFB signaling in both cardiac and dermal fibroblasts [47,48]. Conversely, TGFB also regulates the expression of IL-6 in both smooth muscle cells and lung fibroblasts [49,50]. Another gene, CEBPD, is regulated in response to physiological inducers of ER stress; CEBPD is also a mediator of PERK signaling in chemokine production in melanoma and breast cancer [51].

## 5. Conclusions

In the present study, exposure to TEB resulted in a dose-dependent decrease in MAC-T cell viability in terms of anti-proliferation and apoptotic cell death. The expression of pro-apoptotic proteins, cleaved caspase 3, cleaved caspase 8, and Bax was clearly decreased, and mitochondrial dysfunction was observed in MACT-cells after TEB treatment. Additionally, TEB triggers ER stress through upregulation of Bip/GRP78, PDI, ATF4, CHOP, and ERO1-Lα. Regarding milk protein synthesis, the mRNA levels of LGB, LALA, CSN1S1, CSN1S2, and CSNK were markedly downregulated following TEB treatment. Overall, these results demonstrate that the ER stress–mitochondrial apoptosis pathway is involved in TEB-induced cell death in bovine mammary gland epithelial (MAC-T) cells, which may lead to the downregulation of milk protein synthesis in bovine mammary glands.

## Figures and Tables

**Table 1:** List of primers for qPCR.

Gene	Forward Primer	Reverse Primer
*GAPDH*	5′-GGGTCATCATCTCTGCACCT-3′	5′-GGTCATAAGTCCCTCCACGA-3′
*CHOP*	5′-GCAACGCATGAAGGAGAAAG-3′	5′-AACCATCCGGTCAATCAGAG-3′
*GRP78*	5′-TGGCTGGAAAGTCACCAAG-3′	5′-GTCTGCTGCTTCCTCCTCAC-3′
*ATF4*	5′-GCTGTGGATTGGTTGGTCTC-3′	5′-AGCTCATCTGGCAT-3′
*LGB*	5′-CTTGTGCTGGACACCGACTA-3′	5′-TTGAGGGCTTTGTCGAATTT-3′
*LALA*	5′-AAAGACGACCAGAACCCTCA-3′	5′-GCTTTATGGGCCAACCAGTA-3′
*CSN1S1*	5′-CACTGAGTCAAAGGGAATTAAAG-3′	5′-TGATGGCACTTACAGGAGA-3′
*CSN1S2*	5′-CCTAACAGCCTCCCACAAAA-3′	5′-AGACTGGAGCAGAGGCAGAG-3′
*CSNK*	5′-CCAGGAGCAAAACCAAGAAC-3′	5′-TGCAACTGGTTTCTGTTGGT-3′
*TGFB3*	5′-TCTGGGGCGACTTAAGAAGA-3′	5′-ATTGCGGAAGCAGTAATTGG-3′
*CEBPD*	5′-ATCGACTTCAGCGCCTACAT-3′	5′-TGTGGTTGCTGTTGAAGAGG-3′
*IL6*	5′-AAGCAGCAAGGAGACACTGG-3′	5′-GCCTGATTGAACCCAGATTG-3′

**Table 2:** List of primary antibodies used.

Antibody	Manufacturer	Catalog Number	Dilution (Usage)
Cleaved-caspase3	Cell signaling(Danvers, MA, USA)	#9664S	1:2000 (WB)
Cleaved-capase8	Cell signaling	#8592S	1:2000 (WB)
BAX	Santa Cruz(Dallas, Texas, USA)	Sc-7480	1:2000 (WB)
BIP/GRP78	Cell signaling	#3177	1:2000 (WB)
ERO-La	Cell signaling	#377009	1:2000 (WB)
PDI	Cell signaling	#3501	1:2000 (WB)
β-actin	Santa Cruz	Ab16667	1:2000 (WB)

## Data Availability

Data will be made available on request.
